# Supplementary material for: Maize Apoplastic Fluid Bacteria Alter Feeding Characteristics of Herbivore (Spodoptera frugiperda) in Maize
Source: Microorganisms. 2022 Sep 16;10(9):1850. doi: 10.3390/microorganisms10091850 (PMC9505285; doi:10.3390/microorganisms10091850)
Supplement: Supplementary file 1 [file microorganisms-10-01850-s001.zip › microorganisms-1379939-supplementary.pdf]

microorganisms

MDPI

Supplementary Material

Table S1. Isolation of maize (COH6) leaf and root apoplastic bacteria.

Medium Employed	Leaf Apoplastic Fluid (LAF) Isolates	Root Apoplastic Fluid (RAF) Isolates
100% NA	LAF4, LAF8, LAF9	RAF1, RAF5
50% NA	L6	nd
25% NA	nd	nd
100% LA	LAF5, LAF1	RAF2, RAF6
50% LA	nd	R3
25% LA	nd	nd
100% TSA	LAF7	RAF4
50% TSA	nd	nd
25% TSA	nd	nd
100% R2A	LAF2	nd
50% R2A	nd	nd
25% R2A	nd	nd
Total isolates	8	6

Values are the mean ± standard deviation of experimental data in triplicate. NA, Nutrient Agar; LA, Lysogeny Agar; TSA, Tryptic Soy Agar; R2A, Reasoner's 2A Agar; nd, not detected.

Table S2. Morphological characteristic of isolated apoplastic fluid bacteria.

Isolates	Colony Morphology
LAF 1	Rod, gram positive, buff pigment, irregular, undulated, opaque
LAF 2	Rod, gram negative, creamy, opaque
LAF 3	Cocci, irregular, opaque, gram negative
LAF 4	Rod, gram positive, small colonies, white, irregular, opaque
LAF 5	Rod, gram positive, irregular, opaque
LAF 6	Cocci, gram positive, white, opaque
LAF 7	Rod, gram positive, white, irregular, opaque, filamentous
LAF 8	Rod, gram negative, white, irregular, filamentous
LAF 9	Cocci, gram negative, large colony, white, creamy, yellow
RAF 1	Cocci, gram negative, large colony, white, creamy, opaque
RAF 2	Cocci, gram negative, creamy white, opaque
RAF 3	Cocci, gram positive, small colonies, opaque, creamy
RAF 4	Cocci, gram negative, large colony, creamy, opaque
RAF 5	Rod, gram positive, light yellow, creamy, entire opaque
RAF 6	Rod, gram negative, regular, white, opaque

LAF, Leaf Apoplastic Fluid; RAF, Root Apoplastic Fluid.
